# Endothelin-1 Stimulates Monocytes *in vitro* to Release Chemotactic Activity Identified as Interleukin-8 and Monocyte Chemotactic Protein-1

**DOI:** 10.1155/S0962935194000207

**Published:** 1994

**Authors:** E. Helset, T. Sildnes, Z. S. Konopski

**Affiliations:** 1 Department of Anaesthesiology Institute of Clinical Medicine University of Tromsø Tromsø N-9038 Norway; 2 Department of Experimental Pathology Institute of Medical Biology University of Tromsø Tromsø N-9038 Norway

## Abstract

In the present study we examined whether endothelin-1 stimulation of human monocytes causes release of chemotactic factors. It was found that monocytes released neutrophil- and monocyte-chemotactic activity in a dose- and time-dependent manner in response to ET-1. ET-1 did not show any chemotactic activity by itself. NCA was detected in monocyte supernatants in response to ET-1 (0.01–100 nM) after 1, 4, 8 and 24 h stimulation. MCA was detected only after 24 h stimulation with ET-1 (0.1–100 nM). Preincubation of the monocyte cultures with the lipoxygenase inhibitors nordihydroguaiaretic acid (10^−4^ M) or diethylcarbamazine (10^−9^ M) completely abolished the appearance of NCA and MCA. NCA was neutralized by > 75% using a polyclonal antibody against human interleuktn-8. The ET-1 induced release of IL-8 was confirmed by IL-8 ELISA. A monoclonal antibody against human monocyte chemotactic protein-1 neutralized MCA by > 80%. It is concluded that ET-1 stimulation of monocytes *in vitro* causes release of neutrophil- and monocyte-chemotactic activity identified as IL-8 and MCP-I respectively. An intact lipoxygenase pathway is crucial for this effect of ET-1 to occur.


Mediators of Inflammation 3, 155–160 (1994)

### Endothelin-1 stimulates monocytes *in vitro* to release chemotactic activity identified as interleukin-8 and monocyte chemotactic protein-1

E. Helset,[1,CA] T. Sildnes[1] and Z. S. Konopski[2]

[1]Department of Anaesthesiology, Institute of Clinical Medicine, and [2]Department of Experimental Pathology, Institute of Medical Biology, University of Tromsø, N-9038 Tromsø, Norway

[CA] Corresponding Author

**In the present study we examined whether endothelin-1 stimulation of human monocytes causes release of chemotactic factors. It was found that monocytes released neutrophil- and monocyte-chemotactic activity in a dose- and time-dependent manner in response to ET-1. ET-1 did not show any chemotactic activity by itself. NCA was detected in monocyte supernatants in response to ET-1 (0.01 – 100 nM) after 1, 4, 8 and 24 h stimulation. MCA was detected only after 24 h stimulation with ET-1 (0.1 – 100 nM). Preincubation of the monocyte cultures with the lipoxygenase inhibitors nordihydroguaiaretic acid ($10^{-4}$ M) or diethylcarbamazine ($10^{-9}$ M) completely abolished the appearance of NCA and MCA. NCA was neutralized by > 75% using a polyclonal antibody against human interleukin-8. The ET-1 induced release of IL-8 was confirmed by IL-8 ELISA. A monoclonal antibody against human monocyte chemotactic protein-1 neutralized MCA by > 80%. It is concluded that ET-1 stimulation of monocytes *in vitro* causes release of neutrophil- and monocyte-chemotactic activity identified as IL-8 and MCP-1 respectively. An intact lipoxygenase pathway is crucial for this effect of ET-1 to occur.**

**Key words:** Atherosclerosis, Cytokines, Inflammation, Lipoxygenase, Macrophages

### Introduction

Endothelin-1 is a 21-amino acid peptide, initially isolated from endothelial cells and described as the most potent biological vasoconstrictor known.[1,2] It has recently been shown that ET-1 also might activate monocytes and macrophages, causing release of tumour necrosis factor-α (TNFα), interleukin-1β (IL-1β) and interleukin-6 (IL-6).[3]

When activated, monocytes release several chemotactic factors for monocytes and neutrophils. Some chemotactic factors such as TNFα and leukotriene $B_4$ ($LTB_4$) show chemotactic activity for both monocytes and neutrophils,[4,5] while interleukin-8 (IL-8) has chemotactic activity for neutrophils and lymphocytes, but not for monocytes[6]. Monocyte chemotactic protein-1 (MCP-1) has specific chemotactic and activating activity for monocytes.[7]

To further examine the pro-inflammatory effects of ET-1, human monocytes *in vitro* were stimulated with ET-1 for different time intervals, and the supernatants tested for monocyte- and neutrophil chemotactic activity. To identify the factors responsible for the chemotactic activity, neutralization experiments with antibodies against known chemotactic factors were performed. For comparison, supernatants from ET-1 stimulated human endothelial cells were also tested for chemotactic activity.

### Methods

*Isolation and cultivation of monocytes:* Highly purified monocytes were obtained using the method described previously[8]. Briefly, unseparated mononuclear cells (PBMC) were isolated from human $A^+$ blood buffy coat (The Blood Bank, University Hospital of Tromsø, Norway) by density centrifugation with Lymphoprep (Nycomed Pharma AS, Oslo, Norway). The cells were washed three times with HBSS (Gibco, Glasgow, UK) and resuspended in medium consisting of RPMI-1640 with 100 IU/ml penicillin, 100 μg/ml streptomycin and supplemented with 25% $A^+$ human serum (The Bloodbank, University Hospital of Tromsø). The peripheral blood mononuclear cells (PBMC) were then seeded in 24-well culture plates (Falcon, Becton Dickinson Labware, NJ, USA) at concentration $2 \times 10^6$ cells/well. After incubation at 37°C for 90 min the cell cultures were washed three times with pre-warmed RPMI-1640 to remove non-adherent cells. The monocyte cultures were stimulated with endothelin-1 (ET-1) (Nova Biochem, Laufelingen, Switzerland) immediately. For chemotaxis assay, PBMC resuspended in RPMI-1640 supplemented with 2% human serum, $1 \times 10^6$ cells/ml were used. The purity of the monocyte preparations was assessed morphologically with Wright's stain showing > 80% monocytes. Their viability was > 95% as assessed by Trypan blue exclusion.

©1994 Rapid Communications of Oxford Ltd

*Isolation of human neutrophils:* Neutrophils for use in chemotaxis assay, were isolated from heparinized blood from healthy adult donors by density centrifugation with Polyprep (Nycomed Pharma AS, Oslo, Norway), as described.[8] Contaminating erythrocytes were lysed with 0.2% NaCl for 60 s. The cells were kept on ice, washed and resuspended at $1 \times 10^6$/ml in RPMI-1640 supplemented with 2% human serum and used immediately in experiments. The purity of the neutrophil preparations was > 95% as assessed morphologically with Giemsa staining, and the viability was > 98% tested by Trypan blue exclusion.

*Stimulation of monocyte cultures with ET-1:* The monocyte cultures were stimulated with various concentrations of ET-1 ranging between 0.005 nM and 100 nM for 1, 4, 8 and 24 h. As control, RPMI-1640 instead of ET-1 was used in each experiment. The supernatants were harvested at the indicated time points and stored at –20°C before being assayed for neutrophil- and monocyte chemotactic activity.

To exclude possible contamination with endotoxin, boiling of ET-1 for 30 min, which denaturates the peptide but leaves endotoxin intact, was used in stimulation experiments. In some experiments ET-1 was preincubated with ET-1 antiserum prior to addition to the cell cultures. In addition, supernatants and ET-1 were tested by Endospecy (Seikagaku Kogyo, Tokyo, Japan).

*Preparation of endothelial cell cultures and stimulation with ET-1:* Primary cultures of endothelial cell monolayers (ECM) were prepared from human umbilical veins according to the method of Jaffe *et al.* with slight modifications.[9,10] Endothelial cells, $0.9 \times 10^6$, were grown in medium 199E . . 11 (Gibco Ltd, Middlesex, UK) containing heat-inactivated foetal calf serum (FCS) (Gibco) and 0.2 M L-glutamine (Gibco). The endothelial cell cultures were stimulated with various concentrations of ET-1 ranging between 0.005 nM and 100 nM for 24 h. The supernatants were harvested and analysed for chemotactic activity.

*Chemotaxis assay:* The chemotaxis assay was performed in a 48-well chemotaxis chamber (Neuroprobe Inc., Cabin John, MD, USA) as described previously.[11] The bottom wells of the chamber were filled with 25 μl of supernatant in duplicate or triplicate. A polyvinyl pyrrolidone (PVP) polycarbonate filter (Neuroprobe Inc., Cabin John, MD, USA) with a pore size of 5 μm for monocyte chemotaxis, or a 5 μm PVP free polycarbonate filter[12] for neutrophil chemotaxis assay was placed over the bottom wells. The upper wells were filled with 50 μl of cell suspension. The chamber was then incubated in humidified air in 5% $CO_2$ at 37°C for either 40 min for neutrophil chemotaxis assay or 3 h for monocyte chemotaxis assay. After incubation the chamber was disassembled, and the filter was fixed in 2.5% glutaraldehyde (Merck, Darmstadt, Germany), 0.1 M sucrose, and 0.1 M cacodylate buffer, pH 7.3, for 30 min at room temperature. The filter was then stained with 1% Giemsa (Sigma) for 30 min, and mounted on a glass slide. Cells completely migrated through the filter were counted in 6–12 high power fields (HPF, × 100). Chemotactic response was expressed as the mean number of cells per one HPF in duplicate or triplicate wells. RPMI-1640 was used to determine background migration. Formyl–methionyl–leucyl–phenylalanine (FMLP, Sigma) at a concentration of $10^{-7}$ M, was used as positive control.

*Neutralization studies:* In the neutralization studies, supernatants from monocytes stimulated with ET-1 for different time intervals were incubated with a polyclonal goat anti-human IL-8 antibody (British Biotechnology, UK); or a monoclonal anti-human TNFα antibody (Boehringer Mannheim, Germany); or a monoclonal anti-human MCP-1/MCAF antibody (PeproTech Inc., NJ, USA) for 1 h at 37°C, before being tested for neutrophil and monocyte chemotactic activity. Controls were performed using non-immune serum. Anti-human IL-8 antibody, 1 mg/ml, was used at an initial dilution of 1:75 in the experiments. Anti-human TNFα antibody, 200 μg/ml, was used at an initial dilution of 1:50. Anti-human MCP-1/MCAF antibody, 1 mg/ml, was used at an initial dilution of 1:50 in the experiments. The decrease in chemotaxis was expressed as the percentage decrease in migration after addition of the neutralizing antibody ([before antibody–after antibody] × 100 / [before antibody]).

*IL-8 ELISA:* Supernatants from monocytes stimulated with ET-1 for different time intervals, were analysed for IL-8 content by interleukin-8 ELISA (Quantikine kits, British Biotechnology). A NovaPath™ Mini Reader (BioRad, Richmond, CA, USA) was used in reading of the microplates at $OD_{450}$.

*Effect of lipoxygenase inhibitors on release of NCA and MCA:* The lipoxygenase inhibitors nordihydroguaiaretic acid (NDGA, 50 μM–400 μM, Sigma) or diethylcarbamazine (DEC, 0.62 nM–10 nM, Sigma) was added to the monocyte cultures 5 min before ET-1. The cell cultures were incubated at 37°C for different time intervals as described above, and the supernatants were collected and examined for NCA and MCA. The effect on NCA was examined after 1, 4, 8 and 24 h incubation, while the effect on MCA was examined after 24 h incubation. NDGA or DEC did not affect monocyte viability as tested by Trypan blue exclusion.

*Statistics:* The difference between groups was tested for significance using a Student's *t*-test, or one-way ANOVA supplied with Scheffe's test.[13] In all cases, a

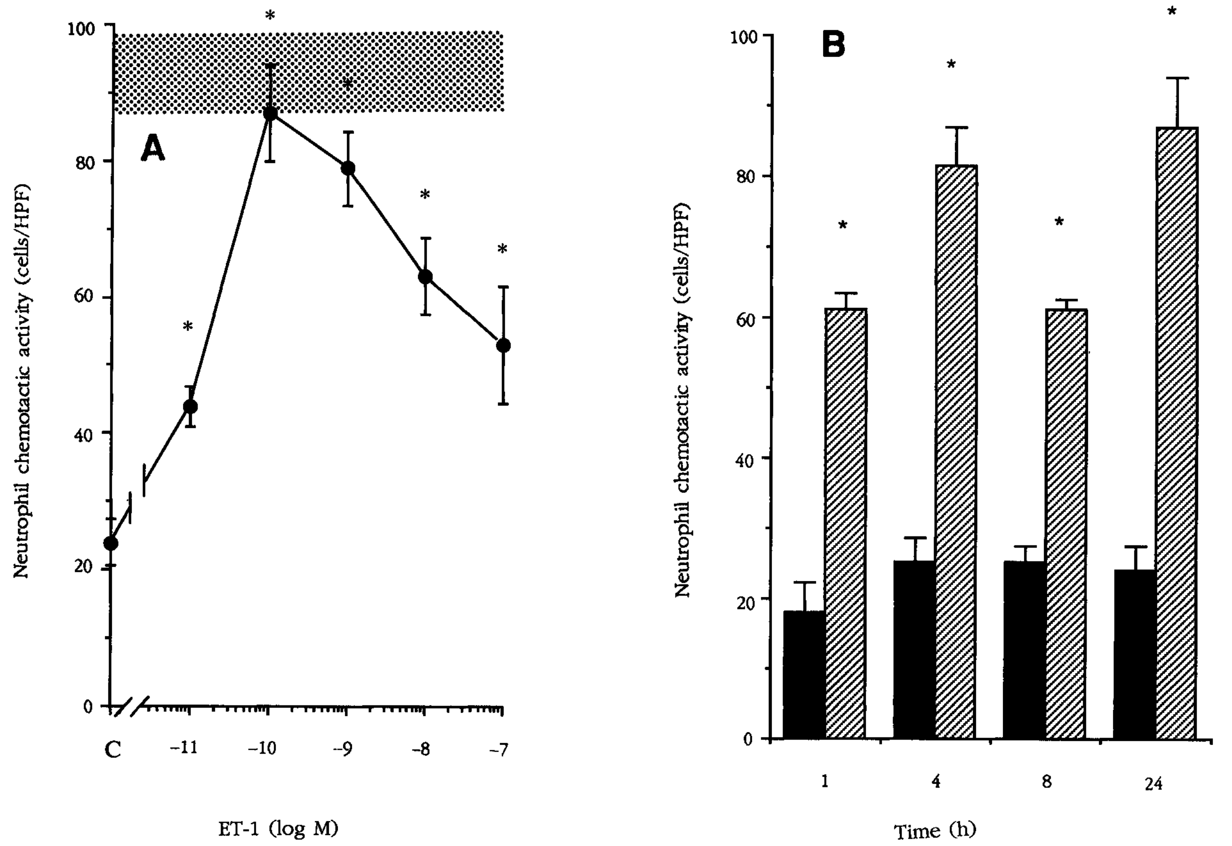

FIG. 1. (A) Dose dependent release of neutrophil chemotactic activity in response to ET-1 from human monocytes after 4 h stimulation. Shadowing represents the response to FMLP $10^{-7}$ M. C: controls, supernatants from monocytes incubated with RPMI-1640 instead of ET-1. (B) Release of neutrophil chemotactic activity in response to 0.5 nM ET-1 as a function of time. ▨, ET-1 stimulated cells. ■, controls. Results are presented as mean ± S.E.M. ($n = 8$). $p < 0.05$ compared with controls.

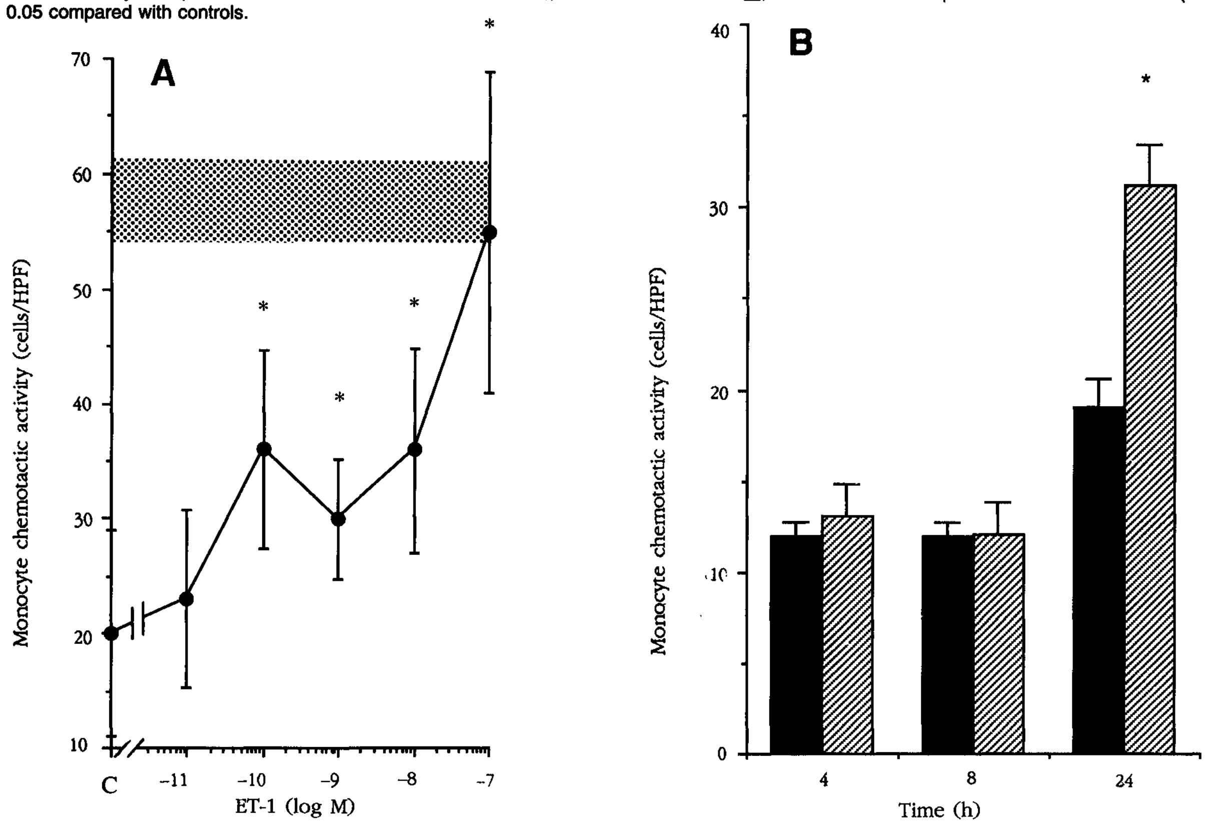

FIG. 2. (A) Dose dependent release of monocyte chemotactic activity in response to ET-1 from human monocytes after 24 h stimulation. Shadowing represents the response to FMLP $10^{-7}$ M. C: controls, supernatants from monocytes incubated with RPMI-1640 instead of ET-1. (B) Release of monocyte chemotactic activity in response to 100 nM ET-1 as a function of time. ▨, ET-1 stimulated cells. ■, controls. Results are presented as mean ± S.E.M. ($n = 8$). $*p < 0.05$ compared with controls.

$p$ value < 0.05 was considered significant. Data in the figures and tables are expressed as mean ± S.E.M.

### Results

*Release of neutrophil (NCA) and monocyte chemotactic activity (MCA) from ET-1 stimulated monocytes:* Monocytes released NCA in response to ET-1 in a biphasic manner as shown in Fig. 1A. The maximum response was measured at 0.5 nM ET-1. This was comparable to the NCA seen in response to FMLP $10^{-7}$ M. ET-1 concentrations lower than 0.01 nM gave no significant increase in NCA. Background migration, in response to RPMI-1640 was 6.2 ± 3.2 neutrophils/HPF. The time course study (Fig. 1B) showed that the release of NCA was significant by 1 h after addition of ET-1, with a maximal response measured after 4 h, and a further increase after 24 h stimulation.

Monocytes released MCA in response to ET-1 in a dose dependent manner as shown in Fig. 2A. The maximum response was seen after stimulation with 100 nM ET-1. This was comparable to the MCA seen in response to FMLP $10^{-7}$ M. Background migration, in response to RPMI-1640, was 7.2 ± 5.1 monocytes/HPF. The time course study showed that the release of MCA was significantly increased in the monocyte supernatants only after 24 h stimulation with ET-1.

No significant amounts of endotoxin were detected in the supernatants or ET-1 used in the experiments. Furthermore, denaturation of ET-1 by boiling, or preincubation of ET-1 with ET-1 antiserum prior to addition to the cell cultures, neutralized the ET-1 induced release of chemotactic activity.

No release of MCA or NCA was observed from ET-1 stimulated endothelial cell cultures compared with background migration (RPMI-1640) (data not shown). ET-1 was not toxic for endothelial cells at any concentration, as assessed by release of lactate dehydrogenase (LDH) (data not shown). No changes in endothelial cell morphology were observed by light microscopic examination.

**Table 1.** Effect of ET-1 on IL-8 release from human monocytes *in vitro*[a]

| Concentration of ET-1 (M) | Concentration of IL-8 (ng/ml) | | |
|---|---|---|---|
| | 1 h | 4 h | 24 h |
| None (controls) | 0.7 ± 0.0 | 0.7 ± 0.0 | 1.4 ± 0.1 |
| $10^{-10}$ | 1.4 ± 0.1 | 2.2 ± 0.0 | 2.4 ± 0.0 |
| $10^{-8}$ | 1.0 ± 0.1 | 2.0 ± 0.1 | 2.8 ± 0.1 |

[a] Supernatants from monocytes were stimulated with ET-1 for different time intervals and examined for their IL-8 content using an IL-8 ELISA kit. The results are presented as mean ± S.D. of triplicate determinations. The results are from one donor, and are representative of three independent experiments with different donors.

*Immunoadsorption of monocyte and neutrophil chemotactic activity:* Incubation with an antibody against IL-8 (dilution 1/100) reduced NCA in the supernatants by > 75% (Fig. 3). The migration after addition of antibody was not significantly different from controls incubated with medium only; 22 ± 3 cells/HPF. Higher antibody concentrations did not increase the inhibition of NCA, while lower antibody concentrations showed a significant reduction in inhibitory effect (data not shown). Release of IL-8 from ET-1 stimulated monocytes was further confirmed using IL-8 ELISA as shown in Table 1.

Incubation with a monoclonal antibody against MCP-1 (dilution 1/200) reduced MCA by > 80% (Fig. 4). The migration after addition of antibody was not significantly different from controls incubated with

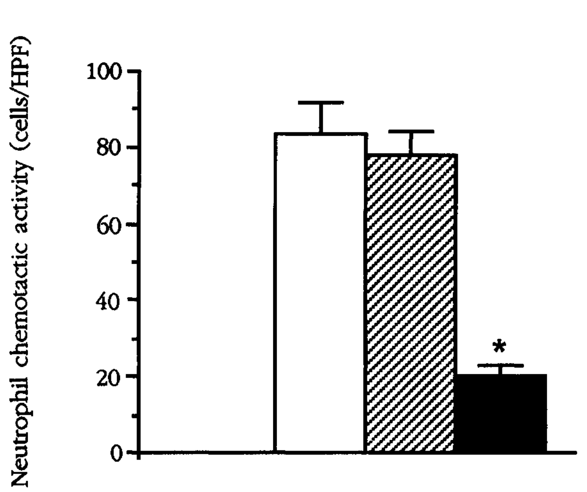

FIG. 3. Anti-human IL-8 antibody neutralized neutrophil chemotactic activity (NCA) in supernatants from ET-1 stimulated monocytes. Samples from ET-1 stimulated cells were treated with control antibody or anti-IL-8 antibody and NCA was assessed. Data represent mean ± S.E.M. ($n = 6$). Open bars: samples; shaded bars: sample plus control antibody; closed bars: sample plus anti-human IL-8 antibody. *$p$ < 0.05 compared with samples not incubated with antibody.

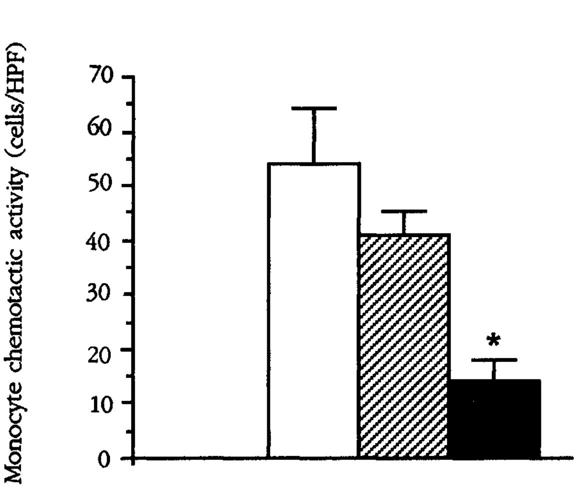

FIG. 4. Anti-human MCP-1 antibody neutralized monocyte chemotactic activity (MCA) in supernatants from ET-1 stimulated monocytes. Samples from ET-1 stimulated cells were treated with control antibody or anti-MCP-1 antibody and MCA was assessed. Data represent mean ± S.E.M. ($n = 6$). Open bars, samples; shaded bars, sample plus control antibody; closed bars, sample plus anti-human MCP-1 antibody. *$p$ < 0.05 compared with samples not incubated with antibody.

**Table 2.** The effect of the lipoxygenase-inhibitors NDGA and DEC on the ET-1 induced release of neutrophil and monocyte chemotactic activity from human monocytes *in vitro*[a]

| | NCA (cells/HPF) | MCA (cells/HPF) |
|---|---|---|
| Controls | 13 ± 4 | 8 ± 2 |
| Sample | 55 ± 6 | 40 ± 7 |
| NDGA, 50 μM | 26 ± 5* | 20 ± 4* |
| DEC, 2.5 nM | 20 ± 6* | 12 ± 5* |

[a] The monocyte cultures were preincubated with NDGA or DEC before ET-1 was added. Controls represent monocyte cultures incubated with RPMI-1640 instead of ET-1. 'Sample' represents the response to 0.1 nM ET-1 and 100 nM ET-1 for release of NCA and MCA, respectively. Results are presented as mean ± S.E.M. ($n = 8$). *$p < 0.05$ compared with sample response. One-way ANOVA supplied with Scheffe's test.

medium, 18 ± 4 cells/HPF. Higher antibody concentrations did not significantly increase the inhibition of MCA, while lower antibody concentrations significantly reduced the inhibitory effect (data not shown). Incubation with an antibody against TNFα showed no neutralizing effect on MCA or NCA (data not shown).

*Effect of lipoxygenase inhibitors on release of monocyte- and neutrophil chemotactic activity:* ET-1 has been shown previously to activate the arachidonic acid cascade system in macrophages.[14] Therefore we wanted to determine the possible contribution of lipoxygenase pathway for the ET-1 induced release of NCA and MCA. As shown in Table 2 NCA and MCA were significantly reduced when the monocytes were incubated with one of the lipoxygenase inhibitors, NDGA or DEC, before ET-1 was added. The reduction was dependent on the dose of lipoxygenase inhibitor used, and was independent of incubation time (data not shown). NDGA and DEC alone did not show any NCA or MCA.

### Discussion

In the present study it is shown that ET-1 functions as a signal molecule for monocytes causing release of chemotactic factors for neutrophils and monocytes. The dose–response pattern for NCA with a maximal response at an ET-1 concentration of $10^{-10}$ M is similar to the dose–response pattern previously observed for release of TNF*a*, IL-1*b* and IL-6 from monocytes and macrophages.[3] Viability tests, however, excluded the possibility that higher concentrations of ET-1 had a toxic effect on the cells. The striking difference in dose-response and the time course for NCA and MCA indicated that different chemotactic factors for neutrophils and monocytes were produced in response to ET-1. This was confirmed in immunoadsorption studies showing that specific antibodies against IL-8 neutralized the neutrophil chemotactic activity, while monoclonal antibodies against MCP-1 neutralized the monocyte chemotactic activity. Although these antibodies neutralized MCA and NCA by > 80% and > 75% respectively, we cannot exclude the possibility that other chemotactic factors are released after stimulation of monocytes with ET-1.

A significant increase in IL-8 secretion was demonstrated at all time-points measured. This time course, with rapidly induced and long-lasting release of IL-8, is in accordance with previous observations on IL-8 release from activated mononuclear phagocytes.[6,15,16] Although monocytes are determined to be the predominant producing cells for IL-8, vascular endothelial cells have also been shown to produce IL-8 when stimulated with lipopolysaccharide or TNF and IL-1.[6] In the present study no NCA is released from endothelial cells in response to ET-1, indicating a disparate regulation of IL-8 release from monocytes and endothelial cells.

The monocyte chemotactic activity, which was neutralized with monoclonal antibodies against MCP-1, was observed in the supernatants only after 24 h stimulation with ET-1. This is in accordance with previous time course studies on MCP-1 release from activated monocytes, showing a maximal response in MCP-1 production after 24 h.[16,17] MCP-1 was recently shown to be responsible for the majority of the chemotactic activity released from vascular endothelium.[18] However, the present results show that ET-1 causes no release of monocyte chemotactic activity from endothelial cells.

Preincubation of the monocytes with the lipoxygenase inhibitors NDGA and DEC, inhibited the release of both monocyte and neutrophil chemotactic activity. ET-1 has previously been reported not to release $LTB_4$ from monocytes,[19] while ET-1 stimulation of alveolar macrophages causes release of arachidonic acid.[14] Evidence is increasing that the lipoxygenase pathway and lipoxygenase products such as $LTB_4$ might play a role of feedback regulators or intracellular messengers for production of cytokines, including IL-8.[20,21] Thus the present results indicate that an intact lipoxygenase pathway is crucial for the ET-1 induced release of IL-8 and MCP-1.

ET-1 has previously been shown to cause a leukocyte dependent increase in microvascular permeability in isolated rat lungs.[22] The mechanism for the ET-1 induced lung injury might be explained by a synergistic action of TNFα, IL-1β and IL-8 from monocytes and tissue macrophages, causing neutrophil recruitment and increased microvascular permeability.[7,10]

Patients with atherosclerosis have increased circulating levels of ET-1, which exhibit a positive correlation with the extension of disease.[23] MCP-1 and IL-8 have also been detected in early vascular lesions in atherosclerosis, predominantly localized to tissue macrophages.[24,25] On this background we suggest that released ET-1 at predilection sites for atherosclerosis,[23,26] might stimulate mono-cytes and/ or tissue macrophages in the vascular wall to release MCP-1 and IL-8, and thus contribute to recruitment of inflammatory cells into the vessel wall causing a chronic inflammatory condition and finally atherosclerosis.[18,23,24]

To conclude, ET-1 stimulation of human monocytes *in vitro* caused release of monocyte and neutrophil chemotactic activity provided that an intact lipoxygenase pathway was present. The neutrophil- and monocyte chemotactic activity were identified as IL-8 and MCP-1, respectively.

### References

1. Yanagisawa M, Kurihara H, Kimura S, *et al.* A novel potent vasoconstrictor peptide produced by vascular endothelial cells. *Nature* 1988; **332**: 411–415.
2. Yanagisawa M, Masaki T. Molecular biology and biochemistry of the endothelins. *TIPS* 1989; **10**: 374–378.
3. Helset E, Sildnes T, Seljelid R, Konopski ZS. Endothelin-1 stimulates human monocytes *in vitro* to release TNF-α, IL-1β and IL-6. *Mediators of Inflammation* 1993; **2**: 417–422.
4. Ming JW, Bersani L, Mantovani A. Tumor necrosis factor is chemotactic for monocytes and polymorphonuclear leukocytes. *J Immunol* 1987; **138**: 1469–1474.
5. Borgeat P, Naccache PH. Biosynthesis and biological activity of leukotriene $B_4$. *Clin Biochem* 1990;**23**:459–468.
6. Leonard EJ, Yoshimura T. Neutrophil attractant/activation protein-1 (NAP-1(Interleukin-8)). *Am J Respir Cell Mol Biol* 1990; **2**: 479–486.
7. Yoshimura T, Leonard EJ. Human monocyte chemoattractant protein-1 (MCP-1). In: Westwick J, Lindley IJD, Kunkel SL, eds. *Advances in Experimental Medicine and Biology. Chemotactic Cytokines.* New York: Plenum Press, 1991; **305**: 47–53.
8. Bøyum A. Isolation of mononuclear cells and granulocytes from human blood. *Scand J Clin Lab Invest* 1968; **97**: 77–89.
9. Jaffe EA, Nachman RL, Becker CG, Minick CR. Culture of human endothelial cells derived from umbilical veins. Identification by morphologic and immunologic criteria. *J Clin Invest* 1973; **52**: 2745–2756.
10. Czervionke RL, Hoak JC, Fry GL. Effects of aspirin on thrombin-induced adherence of platelets to cultured cells from the blood vessel wall. *J Clin Invest* 1978; **62**: 847–856.
11. Wilkinson PC. Micropore filter methods for leukocyte chemotaxis. In: DiSabato G, ed. *Methods in Enzymology. Immunochemical Techniques, Part L: Chemotaxis and Inflammation.* San Diego: Academic Press, 1988; **162**: 38–50.
12. Harvath L, Falk W, Leonard EJ. Rapid quantitation of neutrophil chemotaxis: use of a polyvinylpyrrolidone-free polycarbonate membrane in a multiwell assembly. *J Immunol Methods* 1980; **37**: 39–45.
13. Altman DG. Practical statistics for medical research. London: Chapman & Hall, 1991; 611.
14. Millul V, Lagente V, Gillardeaux O, *et al.* Activation of guinea pig alveolar macrophages by endothelin-1. *J Cardiovasc Pharm* 1991; **17**(Suppl): S233–S235.
15. Ham JM, Kunkel SL, Dibb CR, Standiford TJ, Rolfe MW, Strieter RM. Chemotactic cytokine (IL-8 and MCP1) gene expression by human whole blood. *Immunol Invest* 1991; **20**: 387–394.
16. DeForge LE, Remick DG. Kinetics of TNF, IL-6 and IL-8 gene expression in LPS-stimulated human whole blood. *Biochem Biophys Res Comm* 1991; **174**: 18–24.
17. Cushing SD, Fogelman AM. Monocytes may amplify their recruitment into inflammatory lesions by inducing monocyte chemotactic protein. *Arteriosclerosis and Thrombosis* 1992; **12**: 78–82.
18. Cushing SD, Berliner JA, Valente AJ, *et al.* Minimally modified low density lipoprotein induces monocyte chemotactic protein 1 in human endothelial cells and smooth muscle cells. *Cell Biology* 1990; **87**: 5134–5138.
19. McMillen MA, Huribal M, Kumar R, Sumpio BE. Endothelin-stimulated human monocytes produce prostaglandin $E_2$ but not leukotriene $B_4$. *J Surg Res* 1993; **54**: 331–335.
20. Rola-Pleszczynski M. $LTB_4$ and PAF in the cytokine network. In: Wong Y-K, Serhan CN, eds. *Advances in Experimental Medicine and Biology. Cell–Cell Interactions in the Release of Inflammatory Mediators.* New York: Plenum Press. 1991; **314**: 205–221.
21. Rankin JA, Sylvester I, Smith S, Yoshimura T, Leonard J. Macrophages cultured *in vitro* release leukotriene $B_4$ and neutrophil attractant/activation protein (interleukin 8) sequentially in response to stimulation with lipopolysaccharide and zymosan. *J Clin Invest* 1990; **86**: 1556–1564.
22. Helset E, Kjæve J, Hauge A. Endothelin-1-induced increases in microvascular permeability in isolated, perfused rat lungs requires leukocytes and plasma. *Circulatory Shock* 1993; **39**: 15–20.
23. Lüscher TF, Boulanger CM, Dohi Y, Yang Z. Endothelium derived contracting factors. *Hypertension* 1992; **19**: 117–130.
24. Koch AE, Kunkel SL, Pearce WH, *et al.* Enhanced production of the chemotactic cytokines interleukin-8 and monocyte chemoattractant protein-1 in human abdominal aortic aneurysms. *Am J Pathol* 1993; **142**: 1423-1431.
25. Nelken NA, Coughin SR, Gordon D, Wilcox JN. Monocyte chemoattractant protein 1 in human atheromatous plaques. *J Clin Invest.* 1991; **88**: 1121–1127.
26. Yoshizumi M, Kurihara H, Sugiyama T, *et al.* Hemodynamic shear stress stimulates endothelin production by cultured endothelial cells. *Biochem Biophys Res Comm* 1989; **161**: 859–864.

ACKNOWLEDGEMENTS. The authors thank Dr J. B. Hansen, University of Tromsø, for supply of endothelial cell cultures. This work was supported by the Norwegian Council for Science and Humanities and the Nordic Insulin Fond.

**Received 13 December 1993; accepted in revised form 18 January 1994**
